# The relationship between attachment and depression among college students: the mediating role of perfectionism

**DOI:** 10.3389/fpsyg.2024.1352094

**Published:** 2024-04-15

**Authors:** Fang Fang, Jingwen Wang

**Affiliations:** ^1^School of Humanities and Social Sciences, Anhui Agricultural University, Hefei, China; ^2^College of Arts, Humanities and Social Sciences, Edinburgh College of Arts, University of Edinburgh, Edinburgh, United Kingdom

**Keywords:** collge students, attachment, depression, perfectionism, medating effects

## Abstract

**Objectives:**

Explore the relationship between adult attachment and depression among college students, and the mechanism of perfectionism.

**Study design:**

Questionnaire survey.

**Methods:**

A questionnaire survey was conducted nationwide, totally 313 valid questionnaires were received. The survey collected information such as adult attachment (Adult Attachment Scale, AAS), depression (The Center for Epidemiologic Studies Depression Scale, CESD), and perfectionism (The Frost Multidimensional Perfectionism Scale, FMPS). Then used SPSS26.0 to analyze the collected data.

**Results:**

There were significant differences in perfectionism and depression between secure attachment and insecure attachment in college students. Perfectionism plays a partial mediating role in the relationship between the close-depend composite dimension, anxiety dimension of attachment and depression.

**Conclusion:**

The closeness-dependence dimension of attachment significantly and negatively predicted perfectionism and depression; the anxiety dimension of attachment significantly and positively predicted perfectionism and depression; and perfectionism partially mediated the effect of both the closeness-dependence dimension of attachment on depression and the anxiety dimension of attachment on depression. Attachment can directly affect college students’ depression, but also indirectly through perfectionism.

## Introduction

With the popularization of psychological knowledge and attention to psychological issues from all sectors of society, more and more people see the importance of mental health. Especially to young people who are at a critical stage of their development. The transition from academic to social life at university means that university students face a variety of challenges from the outside world as well as from themselves, such as establishing new interpersonal relationships, solving problems in life alone, and learning more specialized knowledge, making them more prone to psychological problems than other people. This is especially true in the context of the epidemic, which has put university students under even more pressure, not least because of depression, a psychological disorder that has become a common sight in recent years. The study of the mechanisms that lead to depression among university students is of great relevance to address and prevent depression among university students.

Attachment characteristics will also be the subject of this thesis as a necessary factor influencing interpersonal interaction patterns, intimate relationship development and other life issues that important university students need to address. Attachment is the initial connection between children and the world, and through various stages of growth and development, the attachment characteristics of adults can differ from those of children. Individuals with a tendency toward perfectionism are chronically unhappy and therefore also depressed. This study will therefore examine the correlated and internal mechanisms of attachment characteristics, perfectionism, and depression in university students.

## Literature review

Depression is an affective disorder, a psychological condition characterized by low mood, hopelessness, and helplessness (Su et al., 2011). Some studies have shown that the incidence of depression among university students is much higher than that of people in other stages (Ibrahim et al., 2013). College students from rural areas who come to the city for study have a higher risk of depression, which may be related to the inadaptability to the learning or living environment and excessive mental or economic pressure (He et al., 2018). In recent years, some scholars have also compared the detection rate of depression among university students in Guangdong before and after the epidemic, and the data showed that the detection rate of major depression was higher in 2020 than in 2022, with little difference in the detection rate of moderate depression and a higher detection rate of mild depression in 2022 (Li et al., 2022).

People who are depressed often feel sad, depressed, lack interest in activities or things they used to enjoy, fail to experience pleasure in life, have reduced self-confidence, blame themselves, often feel fatigued, and are mentally inactive (Su et al., 2011). Maladjustment can lead to depressive symptoms, depressive episodes, and even life-threatening behaviors such as suicide and homicide (Li, 2018). Depression can also seriously affect the normal life of studying and working of university students. At present, depression is an important global public health problem, and its prevention and control are of great significance (Wang et al., 2020). Therefore, we should pay attention to the study of depression among university students and explore its mechanisms in depth to obtain more effective coping strategies.

Adult attachment refers to an adult’s recollection and reproduction of their early childhood attachment experiences and current evaluation of childhood attachment experiences (Shi and Wei, 2007). Attachment theory suggests that attachment enables individuals to form strong and secure affectional ties with significant others and is one of the main sources of security and a sense of control (Liu et al., 2022). In contrast to infants and children who have a single object of attachment, adults have a much wider range of attachments, including parents, relatives, friends, and lovers. Attachment is a relatively stable structure that manipulates external consciousness, guides relationships with parents, and influences strategies and behaviors in subsequent relationships (Wang et al., 2005). Also, different attachment characteristics and types of attachment are reflected in the individual’s unique interpersonal and intimate relationship management. Establishing and maintaining secure and satisfying social relationships is a basic human need, but the deprivation of this need affects interpersonal relationships in adulthood and may create insecure adult attachment patterns (Mikulincer and Shaver, 2019).

The current available research suggests that people with insecure attachment types are at higher risk of chronic major depression (Li et al., 2019), where the types of insecure attachment are preoccupied attachment and fearful attachment. Conradi and Jonge (2009) have shown that insecure attachment has a very important impact on the generation, development, and maintenance of depressed mood. A study found that individuals with insecure attachment were 3.9 times more likely to suffer from depression than individuals with secure attachment (Shi et al., 2013). Studies of university students have also found that depressive status scores are significantly and positively correlated with attachment avoidance and attachment anxiety scores (Ma et al., 2023).

Perfectionism has been conceptualized as a personality variable that underlies a variety of psychological difficulties. Recently, however, theorists and researchers have begun to distinguish between two distinct types of perfectionism, one a maladaptive form that results in emotional distress, and a second form that is relatively benign, perhaps even adaptive (Bieling et al., 2004). Some studies have found that the development of depression are associated with negative perfectionists’ tendency to focus excessively on mistakes, self-doubt, and expectation criticism (Zhang et al., 2013). It has been suggested that insecure attachment and negative perfectionism are risk factors for the formation of an obsessive-compulsive personality (Xu et al., 2021).

Rice and Mirzadeh (2000) examined the relationship between attachment, perfectionism and depression and showed that subjects with insecure attachment were more likely to be maladaptive perfectionists and to experience more significant depression than those with secure attachment. Research on perfectionism and attachment has also found a significant positive correlation between attachment anxiety dimensions and negative perfectionism in adults (Ma et al., 2010). A study explored the mediating role of perfectionism between attachment and depression, and found that attachment anxiety and attachment avoidance both promote the formation of negative dimensions of perfectionism, which in turn leads to depression (Xin-Chun et al., 2013). A study on the relationship between attachment styles and positive/negative perfectionism, anger, and emotional regulation among college students showed that unsafe attachment styles (avoidance and conflict) can predict negative perfectionism, safe attachment types (avoidance and conflict) can predict emotional regulation, and attachment patterns can determine many behaviors, emotions, and adult relationships, which are necessary for personality growth and mental health foundations (Nader et al., 2017).

## Purpose and hypothesis of the study

The purpose of this study is to explore the mechanism of the relationship between attachment characteristics, depression, and perfectionism among college students.

There are three hypotheses in this study: (1) Attachment characteristics significantly predict depression and perfectionism, (2) Perfectionism significantly predicts depression, and (3) Perfectionism mediates the relationship between attachment characteristics and depression in college students.

## Materials and methods

### Measures

#### Participants

This study used the university student population in different regions as the subjects and used the online distribution method to collect a total of 313 valid questionnaires. There were 130 male students (41.5%) and 183 female students (58.5%); 104 only children (33.2%) and 209 non-only children (66.8%); 32 first year university students; 52 s year students, 54 third year students, 140 fourth year students and 35 postgraduate students.

#### Adult attachment

The revised Adult Attachment Scale (AAS) (Wu et al., 2004) was used to measure attachment characters. The scale was revised and developed by Wili Wu et al. and divided into three subscales of closeness, dependence, and anxiety, each with 6 questions each, for a total of 18 questions. A 5-point scale was used, in which the closeness and dependence subscales were combined into a composite closeness-dependence dimension, which was juxtaposed with the anxiety dimension, with higher scores indicating a stronger characteristic dimension. Based on the mean scores of the two dimensions there are four further types of attachment: secure, preoccupied, rejective and fearful, the latter three being insecure attachments. In the present study, the Cronbach’s alpha coefficients for the closeness-dependence dimension and the anxiety dimension of the scale were 0.774 and 0.866, respectively.

#### Depression

The Center for Epidemiologic Studies Depression Scale (CES-D) (Wang et al., 1999) was used to measure depression, it contains 20 items on a 4-point scale, scored on a scale of 0, 1, 2 and 3, with the higher the score the more depressed they are. Unlike the SDS, the CES-D is not used to clinically judge depressed patients and is therefore more appropriate for the group of university students in this study, focusing on measuring depressed state of mind. In this study, the Cronbach’s alpha coefficient for the scale was 0.902.

#### Perfectionism

The Frost Multidimensional Perfectionism Scale (FMPS) (Zi and Zhou, 2006) was used to measure perfectionism, developed and revised by Zi Fei et al., is divided into 27 entries and five dimensions. The dimensions are organization, concern over mistakes, parental expectations, personal standards, and doubts about action. The organization dimension is positive perfectionism, and the remaining four dimensions represent negative perfectionism. The scale is rated on a 5-point scale, with higher scores indicating a higher tendency toward perfectionism. In this study the Cronbach’s alpha coefficient for the total scale was 0.916 and the Cronbach’s alpha coefficients for each subscale were 0.834, 0.901, 0.842, 0.824 and 0.730, respectively.

### Data analysis

The raw data from the returned questionnaires were processed using SPSS 26.0 software and firstly, the Harman single factor test was used to test for common method bias. The results of the test showed that the variance explained by the first common factor was 22.206%, which was below the threshold of 40%, and therefore there was no serious common method bias in this study. The mediating role of perfectionism in the relationship between attachment characteristics and depression was also tested using Model 4 in the SPSS plug-in Process v3.5.

## Results

### Analysis of the characteristics of attachment, depression and perfectionism among university students

The AAS scores were transformed by combining the closeness-dependence subscale and the dependence subscale into a composite closeness-dependence dimension and calculating the mean closeness-dependence score; the anxiety subscale was used as a separate anxiety dimension and the mean anxiety score was calculated. The mean scores of the two dimensions were compared and categorized. Those with mean scores of closeness-dependence greater than 3 and mean scores of anxiety less than 3 were classified as secure attachment, while the rest were classified as insecure attachment. Insecure attachment was further classified into three different types of attachment: those with a mean score of closeness-dependence greater than 3 and a mean score of anxiety greater than 3 were preoccupied attachment, those with a mean score of closeness-dependence less than 3 and a mean score of anxiety less than 3 were rejective attachment, and those with a mean score of closeness-dependence less than 3 and a mean score of anxiety greater than 3 were fearful attachment. In this study, only 154 (49.2%) were secure attachment, while 54 (17.3%) were preoccupied attachment, 40 (12.8%) were rejective attachment and 65 (20.8%) were fearful attachment.

Of all subjects, 141 (45.05%) experienced some degree of depression.

In the current study, college students’ attachment characteristics, depression and perfectionism did not differ significantly (*p* > 0.05) in the demographic variables of gender, whether they were an only child, and grade level.

The AAS scale scores were transformed to classify the subjects into secure attachment and insecure attachment, 154 in total for the former and 159 in total for the latter, and independent samples *t*-tests were conducted. The results yielded significant differences (*p* < 0.001) between secure attachment subjects and insecure attachment subjects on both depression and perfectionism. As shown in Table 1, subjects with insecure attachment scored significantly higher on depression and perfectionism than those with secure attachment.

**Table 1:** Analysis of differences in depression and perfectionism among college students with different attachment types (*N* = 313).

	Secure attachment (M ± SD)	Insecure attachment (M ± SD)	*t*	*p*
depression	0.70 ± 0.54	1.03 ± 0.55	−5.409	0.000
perfectionism	2.77 ± 0.60	3.27 ± 0.60	−6.809	0.000

### Correlation analysis of attachment characteristics, depression, and perfectionism among university students

Pearson correlation analysis was conducted using SPSS 26.0 on the three variables of attachment characteristics, depression and perfectionism, and the results indicated that all three variables were correlated, which was consistent with the premise of the mediating effect test. The results of the specific correlation analysis are shown in Table 2.

**Table 2:** Correlation analysis of attachment characteristics, depression, and perfectionism among university students (*N* = 313).

	M ± SD	closeness-dependence	anxiety	depression	perfectionism
closeness-dependence	3.32 ± 0.58	1			
anxiety	2.84 ± 0.91	−0.538^**^	1		
depression	2.65 ± 0.87	−0.435^**^	0.369^**^	1	
perfectionism	3.02 ± 0.69	−0.267^**^	0.428^**^	0.361^**^	1

** indicates *p* < 0.01.

The closeness-dependence dimension of attachment was negatively associated with depression and perfectionism, while the anxiety dimension of attachment was positively associated with depression and perfectionism. This means that attachment closeness-dependence scores negatively predict depression and perfectionism, while attachment anxiety dimensions positively predict depression and perfectionism scores. Perfectionism also positively predicted depression.

### A test of the mediating role of perfectionism between attachment characteristics and depression among university students

**Figure 1:**
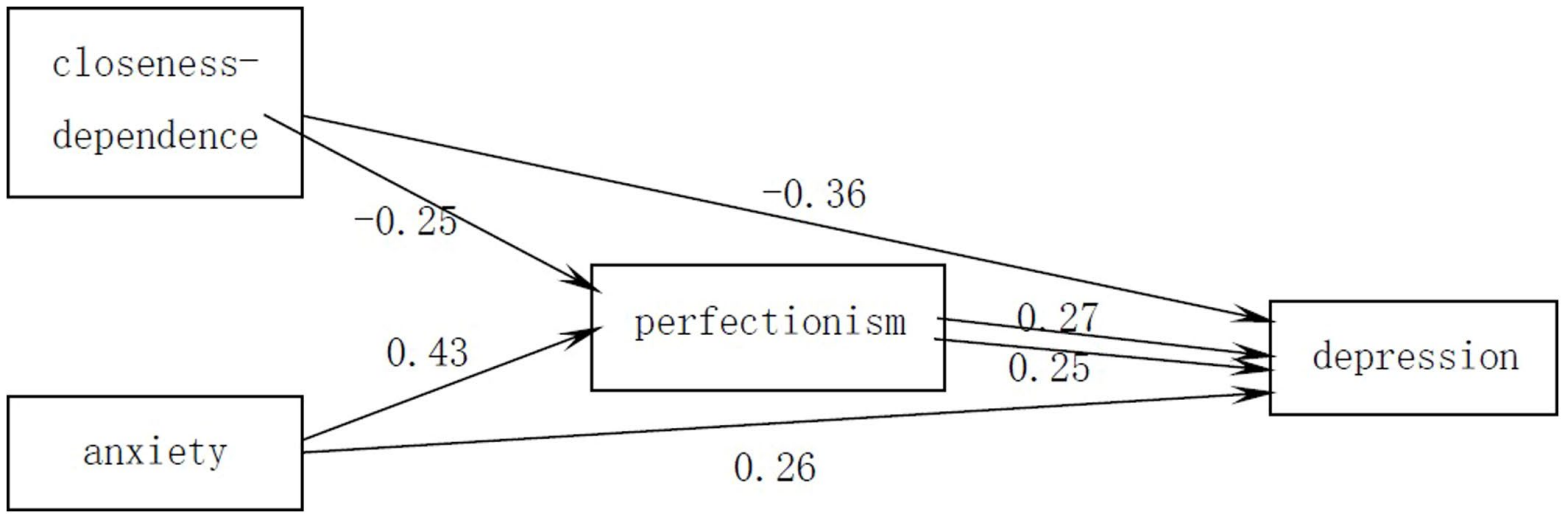
Graph showing the results of the test for the mediating role of perfectionism.

Model 4 in the SPSS plug-in Process prepared by Hayes (Hayes, 2018) was used to test for the mediating effect of perfectionism and the results are shown in Table 3. Before perfectionism was placed as a mediating variable, the closeness-dependence dimension of attachment was a significant negative predictor of depression (*β* = −0.42, *t* = −8.25, *p* < 0.001), the anxiety dimension of attachment was a significant positive predictor of depression (*β* = 0.37, *t* = 6.99, *p* < 0.001). The closeness- dependence dimension of attachment was a significant negative predictor of perfectionism (*β* = −0.25, *t* = −4.88, *p* < 0.001) and the anxiety dimension of attachment was a significant positive predictor of perfectionism (*β* = 0.43, *t* = 8.35, *p* < 0.001). When perfectionism was added as a mediating variable, the closeness-dependence dimension of attachment was still a significant negative predictor of depression (*β* = −0.36, *t* = −6.98, *p* < 0.001) The perfectionism dimension of attachment was also a significant positive predictor of depression (*β* = 0.27, *t* = 5.32, *p* < 0.001); after adding perfectionism as a mediating variable, the anxiety dimension of attachment was still a significant positive predictor of depression (*β* = 0.26, *t* = 4.63, *p* < 0.001), and perfectionism was also a significant positive predictor of depression (*β* = 0.25, *t* = 4.38, *p* < 0.001). The results of the analysis of the mediating effect can be represented in Figure 1.

**Table 3:** Mediating role of perfectionism between attachment and depression (*N* = 313).

Predictor variables	Resulting variables	*R*^2^	*F*	*β*	*t*	95% confidence interval
closeness-dependence	depression	0.18	68.08^***^	−0.42	−8.25^***^	[−0.59, −0.36]
closeness-dependence	perfectionism	0.06	23.83^***^	−0.25	−4.88^***^	[−0.45, −0.19]
closeness-dependence perfectionism	depression	0.25	51.19^***^	−0.36 0.27	−6.98^***^ 5.32^***^	[−0.51, −0.29] [0.14, 0.30]
anxiety	depression	0.14	48.92^***^	0.37	6.99^***^	[0.16, 0.29]
anxiety	perfectionism	0.18	69.80^***^	0.43	8.35^***^	[0.25, 0.40]
anxiety perfectionism	depression	0.19	35.47^***^	0.26 0.25	4.63^***^ 4.38^***^	[0.09, 0.23] [0.11, 0.29]

***indicates *p* < 0.001.

The mediating effects of the attachment closeness-dependence dimension and the anxiety dimension with their respective effect sizes are shown in Table 4.

**Table 4:** Attachment mediated effects and effect sizes.

projects	Effect value	Effect size	95%CI	
			Lower limit	Upper limit
Mediating effect of closeness-dependence—perfectionism—depression	−0.08	16.67%	−0.13	−0.04
The direct effect of closeness-dependence—depression	−0.40	83.33%	−0.51	−0.29
The total effect of closeness-dependence—depression	−0.48	100%	−0.59	−0.36
Mediating effect of anxiety—perfectionism—depression	0.07	30.43%	0.03	0.10
The direct effect of anxiety—depression	0.16	69.57%	0.09	0.23
The total effect of anxiety—depression	0.23	100%	0.16	0.29

The Bootstrap 95% confidence interval for the direct effect of the closeness-dependence dimension of attachment on depression was [−0.51, −0.29], excluding 0, indicating that its direct effect was significant; the Bootstrap 95% confidence interval for the mediating effect of perfectionism between the closeness-dependence dimension of attachment and depression was [−0.13, −0.04], excluding 0, indicating that the mediating effect of perfectionism was significant. The Bootstrap 95% confidence interval for the total effect of attachment closeness -dependence dimension on depression was [−0.59, −0.36], excluding 0, indicating a significant total effect and an effect value of −0.48. Thus, it can be suggested that perfectionism partially mediates the relationship between attachment closeness-dependence dimension and depression, with a mediating effect of 16.67%.

The Bootstrap 95% confidence interval for the direct effect of the anxiety dimension of attachment on depression was [0.09, 0.23], which did not contain 0, indicating a significant direct effect; the Bootstrap 95% confidence interval for the mediating effect of perfectionism between the anxiety dimension of attachment and depression was [0.03, 0.10], which did not contain 0, indicating a significant mediating effect of perfectionism between the anxiety dimension of attachment and depression. The Bootstrap 95% confidence interval for the total effect of attachment anxiety dimension on depression was [0.16, 0.29], excluding 0, indicating a significant total effect with an effect value of 0.23. It can therefore be shown that perfectionism partially mediates between attachment anxiety dimension and depression, with a mediating effect of 30.43%.

## Discussion

### Status of attachment characteristics, perfectionism, and depression among university students

In this study, a total of 45.05% of the university students who participated in the questionnaire experienced some degree of depression, and there were no significant differences in the demographic variables of depression scores in gender, whether they were an only child, or grade level. This also suggests, to some extent, that depression is somewhat prevalent in the university population. In addition, subjects with insecure attachment types were more likely to experience depressive mood than those with secure attachment types. This is in line with the findings of some scholars on adolescent depression and parent–child attachment: secure parent–child attachment reduces the risk of adolescent depression (Lai et al., 2022). The need for excessive attention and commitment is not always met, leading to severe feelings of loss, further doubts, and anxiety about the safety of the relationship (Shaver et al., 2005). College students with insecure attachment types experience more abuse, neglect, separation, and loss in their early years of interaction with their primary caregivers, and such early adverse experiences are often associated with depression (Whiffen et al., 1999). It also points to the important role that attachment plays in the development of adolescents and even university students.

The mechanism by which adult attachment affects depression lies in the internal negative self-model, i.e., the negative evaluation of the self is an important factor in forming depression (Yang, 2005). Individuals with high scores on the anxiety dimension of attachment often fear that the attachment partner will leave them, and one of the reasons for this is a lack of self-confidence and doubt. Because of their negative perceptions of themselves, these groups do not see themselves as worthy of love and therefore feel insecure that they may be abandoned at any time, even if they have established an intimate relationship. Insecure attachment individuals are more likely to have problems in making emotional connections with external objects than secure attachment individuals, e.g., individuals with preoccupied attachment (high closeness-dependence, high anxiety) have a stronger desire to connect with others and a high level of insecurity about existing connections, so they often seek approval from others, are completely focused on others, are dependent on others, easily lose themselves and are in constant fear of relationship breakdown (Zhang, 2021).

In this study, perfectionism was also scored significantly higher in insecure attachment individuals than in secure attachment individuals. Some scholars believe that doing things perfectly and not making mistakes is a means of survival for children who grow up in harsh, punishing, harsh or even abusive families to avoid unnecessary harm or enhance their control over uncertain environments (Flett et al., 2012). Maladaptive perfectionism has overly idealistic expectations and ideas about real life, thinking that they can easily achieve success or achieve goals, often ignoring objective conditions, and often feeling lost and inadequate in the process (Rice and Ashby, 2007). One of the characteristics of perfectionism is that they always set much higher goals for themselves and too difficult to achieve them, so perfectionistic individuals are chronically unfulfilled and dissatisfied with themselves. The insecure attachment individuals’ negative evaluation of self also contributes to the pursuit of high goals. At the same time, prolonged failure to achieve goals can lead to new negative evaluations of the self, which are likely to be accompanied by devaluation of the self, forming a pattern of internal evaluations that can lead to depression. Researcher found that perfectionists’ rigid core thinking produces low levels of unconditional self-acceptance, accompanied by low self-liking and a low sense of self-competence, which in turn leads to depression (Scott, 2007). The Beck depression model suggests that negative notions of self and others increase an individual’s susceptibility to depression (Kernis et al., 1991). It has been shown that maladaptive perfectionist individuals act with excessive consideration for the correctness of their behavior, hesitate to act and fear failure, and are more likely to adopt negative coping styles in stressful situations, leading to anxiety, depression, and other negative emotions (Zhang et al., 2014).

### The mediating role of perfectionism between attachment and depression

The results of the analysis of the mediating role of perfectionism found that the direct effects of the closeness-dependence dimension and the anxiety dimension on depression were significantly weakened after perfectionism was introduced between attachment characteristics and depression as the mediated variable, indicating that perfectionism partially mediates the relationship between both the closeness- dependence composite dimension of adult attachment and the anxiety dimension and depression, meaning that university students’ attachment can directly affect their depression levels, as well as indirectly affect depression levels by influencing perfectionist tendencies.

The closeness-dependence dimension of attachment reflects the degree to which individuals are comfortable in intimate relationships and assess whether they have someone to rely on when needed (Zhang et al., 2014). Individuals with higher levels of comfort in intimate relationships are more likely to feel satisfied and therefore less likely to have negative evaluations of themselves. As shown in the study, scores on the closeness-dependence dimension of attachment were significantly and negatively correlated with scores on perfectionism and depression. It has been found that individuals with higher levels of attachment anxiety have lower levels of emotion expression (Yao and Zhao, 2021), and that the prolonged lack of emotional expression can easily lead to depression. Insecure attachment individuals have negative attitudes toward interpersonal interactions and have less social support than other people, making it more difficult for them to get out of difficult situations when they have negative life events or encounter difficulties and setbacks.

The results of this study show that the closeness-dependence dimension of attachment is negatively related to perfectionism, while the anxiety dimension of attachment is positively related to perfectionism, meaning that individuals with low closeness-dependence and high anxiety in intimate relationships, i.e., Those who do not adapt well to intimate relationships or who have not experienced success in previous intimate relationships, are prone to perfectionism. Perfectionists are also overly concerned with their own behavior and performance. This group believes that they must be complimented and praised by others, and they show a desire for attention and a fear of being rejected by others (Stoeber et al., 2017). This is why these people tend to experience disappointment in their daily lives and in their interpersonal interactions, resulting in a decline in self-confidence, a decrease in interest and depression.

### Implications of the research

This study investigates the mechanisms by which attachment affects depression in university students and has implications for the prevention and intervention of depression in university students. Firstly, it can draw attention to the importance of building secure attachments and creating a good family environment from early childhood to avoid the risk of depression early. At the same time, for attachment characteristics that are difficult to change early in the formative years, it is possible to intervene in depression by changing the mediating variables in this study, guiding individuals to set reasonable goals, and taking more encouraging measures to improve their self-esteem.

### Limitations of the research

This study also has certain limitations, firstly, from the research method, the method of questionnaire collection adopted in this study is online collection method, thus there are limitations in controlling the confounding variables. Moreover, the sample size of this study is still not sufficiently random in terms of grade and major, so there may be some errors. Finally, as perfectionism only partially mediates the results from this study and has a low effect size, there are many potential influences in related areas that need to be further explored.

## Conclusion

There were significant differences in perfectionism and depression between secure attachment and insecure attachment in university students, with insecure attachment individuals scoring higher in perfectionism and depression than secure attachment individuals.

The closeness-dependence dimension of attachment significantly and negatively predicted perfectionism and depression; the anxiety dimension of attachment significantly and positively predicted perfectionism and depression; and perfectionism partially mediated the effect of both the closeness-dependence dimension of attachment on depression and the anxiety dimension of attachment on depression.

## Data availability statement

The raw data supporting the conclusions of this article will be made available by the authors, without undue reservation.

## Ethics statement

The studies involving humans were approved by the Ethics Committee of Anhui Agriculture University. The studies were conducted in accordance with the local legislation and institutional requirements. The participants provided their written informed consent to participate in this study.

## Author contributions

FF: Conceptualization, Supervision, Writing – review & editing, Funding acquisition, Formal analysis. JW: Writing – original draft, Data curation, Methodology.
